# Swifts Form V-Shaped Wings While Dipping in Water to Fine-Tune Balance

**DOI:** 10.3390/biomimetics9080457

**Published:** 2024-07-26

**Authors:** Shuangwei Cui, Zhongjun Peng, Hua Yang, Hao Liu, Yang Liu, Jianing Wu

**Affiliations:** 1 School of Aeronautics and Astronautics, Sun Yat-sen University, Shenzhen 518107, China; cuishw@mail2.sysu.edu.cn (S.C.); pengzhj5@mail2.sysu.edu.cn (Z.P.); 2 School of Advanced Manufacturing, Sun Yat-sen University, Shenzhen 518107, China; maochong3999@163.com; 3 Key & Core Technology Innovation Institute of the Greater Bay Area, Guangzhou 510535, China; liuhao@kctii-gba.com; 4 State Key Laboratory of Biocontrol, School of Ecology, Sun Yat-sen University, Guangzhou 510275, China

**Keywords:** swift, water dipping, wing morphing, motion stability

## Abstract

Swifts, a distinctive avian cohort, have garnered widespread attention owing to their exceptional flight agility. While their aerial prowess is well documented, the challenge swifts encounter while imbibing water introduces an intriguing complexity. The act of water uptake potentially disrupts their flight equilibrium, yet the mechanisms enabling these birds to maintain stability during this process remain enigmatic. In this study, we employed high-speed videography to observe swifts’ water-drinking behavior. Notably, we observed that the swift adopts a dynamic V-shaped wing configuration during water immersion with the ability to modulate the V-shaped angle, thereby potentially fine-tuning their balance. To delve deeper, we utilized a three-dimensional laser scanner to meticulously construct a virtual 3D model of swifts, followed by computational fluid dynamics simulations to quantitatively assess the mechanical conditions during foraging. Our model indicates that the adoption of V-shaped wings, with a variable wing angle ranging from 30 to 60 degrees, serves to minimize residual torque, effectively mitigating potential flight instability. These findings not only enhance our comprehension of swifts’ flight adaptability but also hold promise for inspiring innovative, highly maneuverable next-generation unmanned aerial vehicles. This research thus transcends avian biology, offering valuable insights for engineering and aeronautics.

## 1. Introduction

The flight capacity of swifts is characterized by exceptional speed and agility, frequently marked by swift banking maneuvers and adept exploitation of prevailing winds and atmospheric currents [1,2]. These avian denizens are renowned for their astonishing migratory behaviors, embarking on journeys spanning thousands of miles annually in search of favorable climes for nesting and roosting [3]. During these extensive migrations, swifts exhibit the remarkable ability to sustain continuous flight for months on end, foregoing rest stops—a feat that establishes them as among the most accomplished long-distance avian migrants; some individuals have been documented to cover distances exceeding 10,000 miles in a solitary migration [4,5]. Throughout these extensive journeys, swifts often maintain high altitudes, propelling themselves at speeds of up to 100 km/h to optimize the efficiency of their voyage [4,6]. While water is undoubtedly indispensable for sustaining metabolic functions during their protracted flights, the specifics of how these birds acquire and ingest water have remained largely uncharted territories in scientific inquiry.

Birds exhibit a diverse array of feeding behaviors, including dipping, capillarity hysteresis, aerial drinking, and rainwater collection [7]. Among these, the most prevalent water feeding pattern observed in birds involves the act of dipping their beaks into water and swiftly retracting them while walking along water edges. Take the Apus apus pekinensis, a migratory bird species, as an example. These birds primarily inhabit tall buildings and structures, with limited engagement in ground-based activities [8,9]. This behavior can be attributed to their unique biological adaptations and ecological preferences. The pekinensis species, characterized by its large wingspan, compact body, and short legs, prefers nesting in elevated urban settings [10]. Their physical traits and flight style are conducive to aerial activities. They adeptly seize flying insects mid-flight and even gather water from the air to fulfill their requirements [11]. Consequently, they rarely rely on alighting on the ground to seek sustenance or water sources [8,12]. Similar to other aerial insectivorous birds, swifts have developed specialized drinking behaviors to procure water while being airborne [13]. In the case of swifts, they glide above water surfaces and employ their lower mandibles to scoop up water [7]. Remarkably, there are scarce reports of pekinensis swifts losing stability in motion or accidentally plummeting into the water, and the mechanisms underlying such occurrences remain elusive. For swifts in flight, the act of dipping their beaks into water for sustenance presents a distinctive amalgamation of aerodynamics and fluid dynamics challenges. Maintaining equilibrium while accommodating the introduced perturbations from fluid dynamics poses a formidable task, as the system necessitates achieving both force and torque equilibrium.

Comprehending the underlying mechanisms governing the drinking behavior of swifts holds significant importance as it illuminates the ways in which animals adapt to the interface between air and water. In particular, an exploration into the correlation between the V-shaped wing position and stability during the drinking process offers valuable insights into the adaptations that allow swifts to drink while in flight, all without compromising their equilibrium. Moreover, delving into swifts’ drinking behavior could yield broader implications, enhancing our understanding of the behaviors exhibited by other aerial insectivorous birds and array of adaptations that they have evolved to meet the distinct demands of their lifestyles [14]. In comparison with their avian counterparts like hirundines, swifts display lower maneuverability and are less adept at maintaining lower flight speeds [15,16]. These creatures predominantly inhabit the skies, occasionally engaging in activities such as mating and roosting at elevated aerial heights [10,17,18]. Given their elongated wingspan and diminutive legs, a swift that happens to descend to the ground would encounter difficulties in regaining flight, often necessitating substantial effort [19,20,21].

The Apus apus pekinensis, distinguished by its adept aerial abilities and swift motions, is a diminutive and graceful avian species [22,23,24]. Possessing a sleek and streamlined physique, this bird showcases a wingspan measuring approximately 40–45 cm and a weight ranging between 30 and 40 g [25,26]. Its ventral regions exhibit a slightly lighter hue compared with the dorsal surfaces encompassing its back and wings, while its tail is short and distinctly forked. Additionally, the pekinensis boasts a relatively petite head with a rounded configuration, complemented by a succinct, pointed beak [27,28]. These distinct traits collectively equip the species for its aerial lifestyle and empower it to flourish across a diverse spectrum of environments.

## 2. Materials and Methods

### 2.1. Image Processing

**Figure 1:**
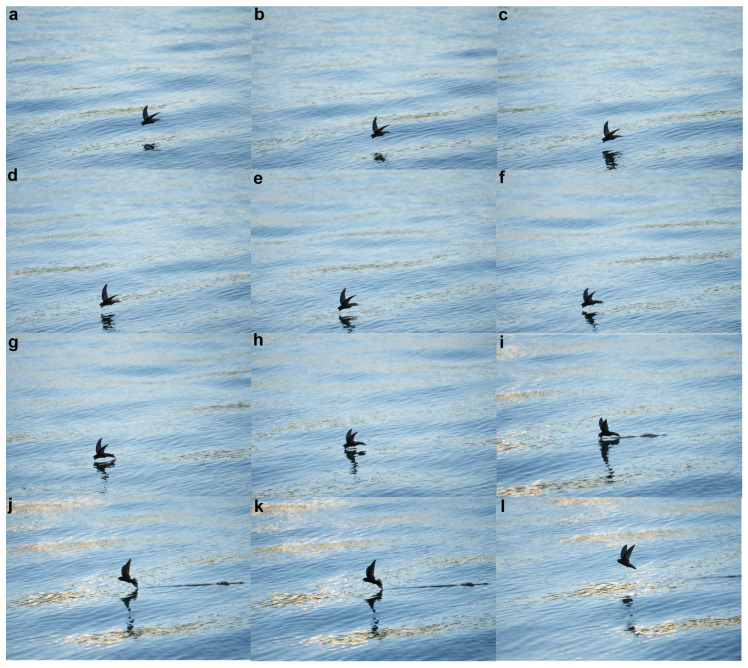
Key frame sequences of the swift’s drinking process.

Initially, we documented the pivotal moments of the swift engaging in water surface feeding, employing a camera (ILCE-7RM4, Sony, Tokyo, Japan) to capture the sequence. The filming of the swifts was carried out by a senior swift expert using a high-speed camera with a frame rate of 3200 frames per second and resolution of 8640 × 5760. The locations where swifts choose to dip water are highly random, and the drinking process is extremely brief, typically on the order of milliseconds. This makes it extremely difficult to manually capture the swift’s drinking process as it requires the photographer to be very familiar with the swift’s habits and usually involves using a fixed camera position. We obtained a total of 13 sets of key frame sequences for the swift’s drinking process. Figure 1 is one of them, with subfigures h, i, and j being selected as key frames to divide the drinking stages of the swift. Please refer to the Supplementary Materials for the remaining 12 sets of key frame sequences. Subsequently, these images were amalgamated to generate a comprehensive video illustrating the complete feeding process. This footage encompassed various stages, encompassing the swift’s navigation across the water surface, the intricate act of beak-immersed water dipping while in flight, and the subsequent maneuvering of the body to disengage from the water’s surface. Subsequently, we meticulously selected crucial images representative of each of these processes, which were then subjected to meticulous analysis. To assess the wing’s morphology, we utilized ANSYS SpaceClaim (version 2022R1), a powerful software tool renowned for its precision in geometric analysis and modeling.

### 2.2. 3D Scans

**Figure 2:**
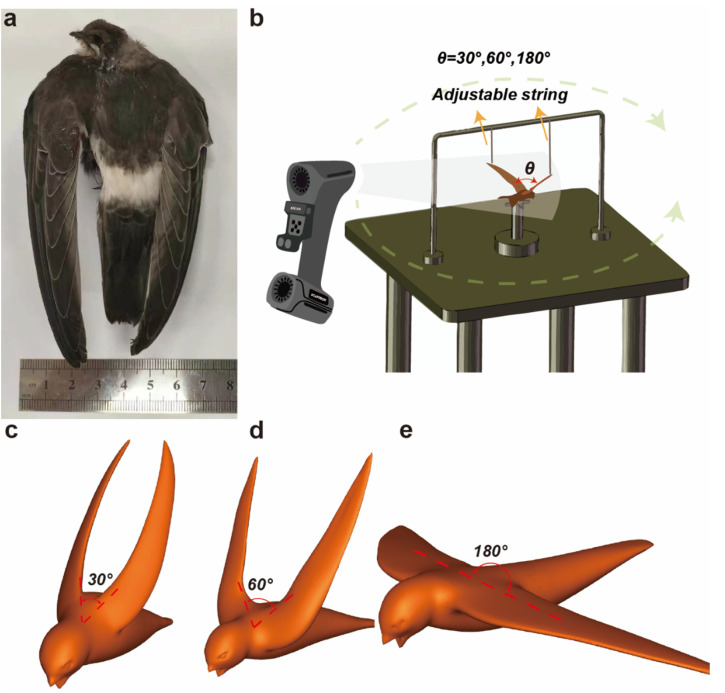
Models of swifts with varying wing angles using the 3D laser scanning technique, along with the experimental setup diagram for the laser scanning experiment. (**a**) The samples of *A. pacificus* used in our experiment. (**b**) Experimental procedure for swift placement and 3D laser scanning. In our experimental setup, the swift was carefully positioned on the experimental platform, ensuring optimal wing angles, and securely immobilized using a flexible apparatus. Subsequently, a $360^{\circ }$ comprehensive scanning of the swift was performed using a 3D laser scanner, resulting in the acquisition of point cloud data representing the morphology of the swift. (**c**) $\theta =30^{\circ }$. (**d**) $\theta =60^{\circ }$. (**e**) $\theta =180^{\circ }$.

The swift samples are frozen specimens obtained from the BioMuseum (Sun Yat-sen University). To acquire the geometric model of the swift, we built our own scanning platform. We then adjusted the swift into a predetermined posture and fixed it on the platform. We employed a 3D laser scanner (KSCAN-MAGIC, Scantech, Stavanger, Norway) to conduct a detailed scan of the swift specimen, as depicted in Figure 2a. For our experimentation, we fabricated two distinct swift models: one with wings set in a flat configuration and the other featuring wings arranged in a V-shape, with the included angle termed as θ (Figure 2b). Subsequently, we transferred the acquired scan data to the FreeFoam software platform, a versatile tool renowned for its capabilities in generating intricate 3D models. Following this, the frozen swift specimen was allowed to thaw to room temperature and was placed upon a testing apparatus characterized by mobile linkages and a supporting framework. This configuration permitted the adjustment of wing angles to values of $30^{\circ }$, $60^{\circ }$, and $180^{\circ }$ (as shown in Figure 2b).

Employing the 3D laser scanner once more, we proceeded to scan the swift specimen, yielding a comprehensive set of point cloud data. These datasets were subsequently imported into FreeFoam for meticulous processing, leading to the creation of streamlined body and wing representations for the swift at wing angles of $30^{\circ }$ (Figure 2c), $60^{\circ }$ (Figure 2d), and $180^{\circ }$ (Figure 2e), respectively.

### 2.3. CFD Simulation

**Figure 3:**
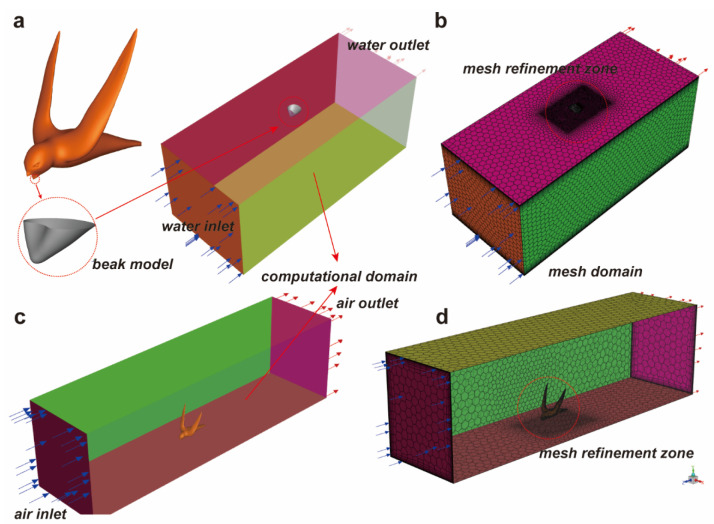
CFD model. (**a**) The geometric model of the swift, generated using ANSYS SpaceClaim, and the model of the beak submerged in water, generated using Boolean operations (left); the computational domain was used to solve for the forces exerted on the beak by water (right). (**b**) The polyhedral mesh of the computational domain employed for solving the forces exerted on the beak by water is refined in regions where there are significant variations in physical quantities near the beak. A boundary layer mesh is generated on the surface to capture the flow characteristics. (**c**) The computational domain utilized for solving the forces exerted on the swift by air involves positioning the swift on the lower base (water surface) to simulate realistic drinking scenarios. (**d**) The polyhedral mesh of the computational domain employed for solving the forces exerted on the swift by air is refined in regions where there are significant variations in physical quantities around the swift. Additionally, a boundary layer mesh is generated on the wall surface to capture the flow characteristics.

The geometric model of the swift, along with its beak submerged in water, was imported into ANSYS SpaceClaim (version 2022R1) for subsequent geometric refinement. Through the utilization of Boolean operations, a comprehensive and defect-free geometric representation was obtained, ensuring the seamless integration of the swift’s form with the submerged beak (as illustrated in Figure 3a). This meticulous procedure aimed to eradicate any inconsistencies in the imported model’s surfaces. Upon verifying the model’s integrity, a bounding cube was generated to enclose both the geometric model of the swift and the submerged beak. This cube delineated the region situated between the inner surface of the cube and the outer surface of the geometric model as the outflow computational domain (as shown in Figure 3a,c). Furthermore, each surface of the model was assigned a reference designation, facilitating subsequent operations.

The geometric model was subsequently imported into ANSYS Fluent Meshing (version 2022R1) for meshing procedures. A sequence of steps was followed, starting with the generation of high-quality surface meshes for the model. This was succeeded by the creation of equally high-quality polyhedron and prism layer meshes, relying on the foundation provided by the surface meshes (depicted in Figure 3b,d). Notably, regions enveloping the swift and its beak were subjected to mesh refinement, ensuring that the calculation outcomes accurately captured intricate details of both the swift’s surface and its immediate vicinity. To account for the wall effect, a boundary layer grid was meticulously applied to the surfaces of the swift model and its beak. This approach was instrumental in capturing precise information regarding the physical properties of the wall flow, contributing to a more comprehensive understanding of the phenomenon under investigation.

### 2.4. Computation Settings

We conducted computational fluid dynamics (CFD) analysis on the swift model using ANSYS Fluent (2022R1). Initially, steady-state simulations were performed using a double-precision pressure-based solver. Considering the transient nature of the drag experienced by the swift model, which may not converge to a single value, a transient solver was initially employed to compute the drag force. However, after multiple simulations, it was observed that the drag force converges satisfactorily to a stable value, prompting the transition to a steady-state solver for subsequent computations. While the standard k−ϵ model combined with standard wall functions is commonly used in engineering CFD simulations and deemed suitable for our operational conditions, we opted for the SSTk−ω model due to its improvements over the k−ϵ model. This model combines the strengths of both k−ϵ and k−ω models, providing enhanced prediction of turbulent behavior in the boundary layer and improving accuracy near the wall, particularly in the presence of adverse pressure gradients. The SSTk−ω turbulence model is a two-equation eddy viscosity model commonly employed in various aerodynamic applications [29,30]. When designing the computational domain, we specifically considered the impact of the boundaries on the results. We controlled the blockage ratio to around 5% to prevent unreliable computational results due to an excessively high blockage ratio. For inlet boundary conditions, a velocity inlet condition was prescribed, and the turbulence intensity and turbulence viscosity ratio were defined to characterize the inlet turbulent state. Considering relatively stable atmospheric conditions during swift flight with weak turbulence, the turbulence intensity was set to 1% and the turbulence viscosity ratio was set to 1. A pressure outlet boundary condition was employed at the domain exit. The backflow turbulence intensity and turbulence viscosity ratio were set as identical to the inlet conditions, although no backflow phenomenon was observed during computations; thus, these settings did not influence the results. The side walls and top wall were set as no-slip wall boundary conditions. The water surface was set as a slip wall boundary. In our calculations, we also tried setting the side walls and top wall as symmetry boundary conditions and found that the results were the same as with the previously used boundary conditions. This consistency is likely due to our computational domain being large enough, so the wall effects of the side walls and top wall do not significantly impact the forces on the swifts. The computational setup included a pressure–velocity coupling algorithm. Pressure discretization utilized a second-order scheme, while velocity, turbulent kinetic energy, and dissipation rate were discretized using a second-order upwind scheme. Specific mesh details were described in the section on grid independence verification.

### 2.5. Mesh Independence

**Figure 4:**
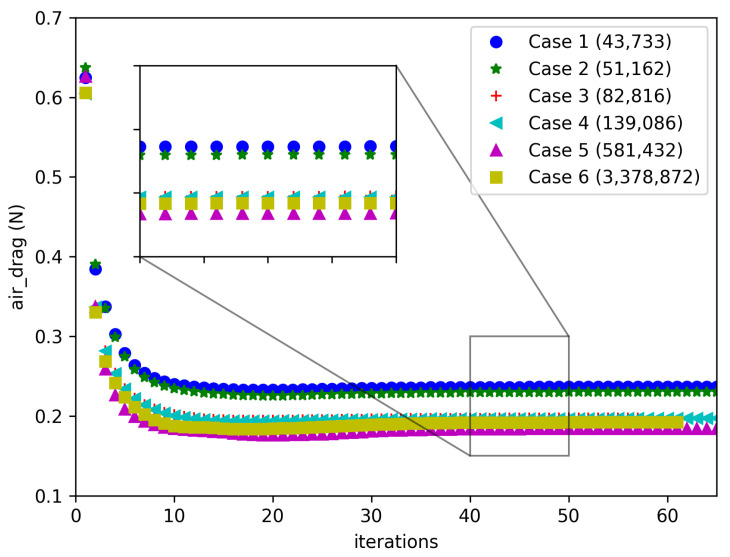
The variation of air drag experienced by the swift with iterations for six grid systems. We used six grid systems with varying resolutions to compute the air drag experienced by the swift model. From Case 1 to Case 5, as the number of grid cells increased, the calculated drag values gradually decreased. In Case 6, further increasing the number of grid cells resulted in the drag value remaining relatively unchanged. We identified the grid-independent solution for the drag and completed the grid independence verification process.

To ensure the accuracy of our computational results and the reliability of our conclusions, we conducted a grid independence study on the drag force experienced by the swift model. We employed six different grid systems and observed the variation in drag force across these grids. Prior to grid generation, to capture fine near-wall flow details, we conducted preliminary calculations to set the first cell height on the wall at approximately 1 mm, ensuring a $y^{+}$ value within 30. Consequently, for the boundary layer grid division, we set the first cell height to 1 mm with a growth rate of 1.2, totaling 5 layers of boundary layer grid. We endeavored to maintain a consistent level of resolution in boundary layer grid refinement across the six grid systems. Additionally, we implemented a locally refined mesh around the swift model to enhance local grid resolution. By varying the mesh sizes within this refinement region, we generated six grid systems with total cell counts of 43,733; 51,162; 82,816; 139,086; 581,432; and 3,378,872. Figure 4 illustrates the variation of drag force on the swift model with iteration steps for different grid numbers. The converged drag values for Case 1 to Case 6 are 0.2365, 0.2301, 0.1976, 0.1970, 0.1842, and 0.1921, respectively. It is evident that the drag force converges to a fixed value as iterations progress, with slight differences being observed among the results from different grid numbers. As the number of cells increases, the drag force gradually decreases. This observation indicates that with fewer cells, there may be deviations in drag force calculations. For instance, the drag force consistently decreases as the cell number increases from 43,733 to 581,432, albeit not dramatically. This suggests that, under the condition of well-resolved boundary layer grids, using relatively sparse meshes may still yield drag force calculations within acceptable ranges.

However, when increasing the grid number from 581,432 to 3,378,872 cells, the drag force results show an increase. This indicates that further increasing the grid number does not necessarily lead to a continued reduction in drag force but rather introduces numerical fluctuations within a small range, which is a normal computational artifact. We identified the correct drag force value within this range. Considering that the computational time with 3,378,872 cells was deemed acceptable, we ultimately selected this mesh configuration for subsequent computations. This approach ensures robustness in our computational findings and enhances confidence in the accuracy of our conclusions regarding the drag force experienced by the swift.

## 3. Results

### 3.1. Feeding Behavior

**Figure 5:**
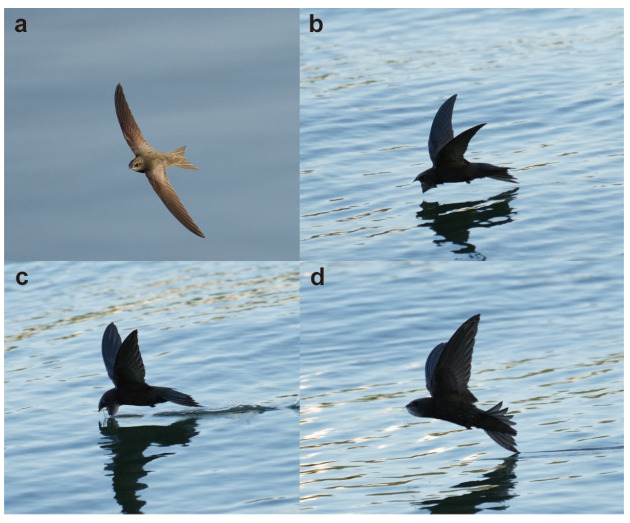
Feeding behavior of a swift on water. (**a**) A cruising Apus apus pekinensis. (**b**) In the initial stage for feeding, the swift prepare for feeding water, with their wings forming a V shape. (**c**) The second stage encompasses the drinking phase of the swift, during which their wings maintain a distinctive V-shaped configuration. (**d**) During the third stage, the bird accomplishes water consumption and subsequently takes off from the water surface.

Utilizing a high-speed camera, we meticulously observed the drinking behavior of the Apus apus pekinensis (Supplementary Materials), a process that can be broadly categorized into three distinct stages, as depicted in Figure 5. During the initial stage, the swift adeptly navigates at a specific altitude above the water surface, actively seeking an optimal location for its drinking activity. The subsequent stage involves the bird initiating a descent towards the water surface. As the swift progressively approaches the water, its wings gradually adopt a V-shaped configuration, maintaining this posture as it elegantly glides above the water to execute the drinking maneuver. Notably, throughout this phase, the swift’s beak penetrates the water’s surface while its rapid movement generates a strong water flow impact. This impact force may potentially prompt the bird to tilt forward. The formation of the V-shaped wings at a specific angle during this juncture likely serves to stabilize the swift’s body posture, contributing to its balance. In the concluding stage, the swift successfully completes its drinking routine, subsequently adjusting its flight trajectory and executing an upward turn. These three stages collectively outline the intricate sequence of events encompassing the swift’s drinking behavior. It is worth noting that, as shown in Figure 5, the posture of the swift’s tail changes during the drinking process. We speculate that these adjustments in tail posture may also help maintain body balance. In our modeling process and numerical simulations, the swift’s tail was kept horizontal, and we did not consider the impact of tail posture changes on balance. We need to clarify this point. In future research, we may conduct an in-depth study on this phenomenon.

### 3.2. Force Analysis

**Figure 6:**
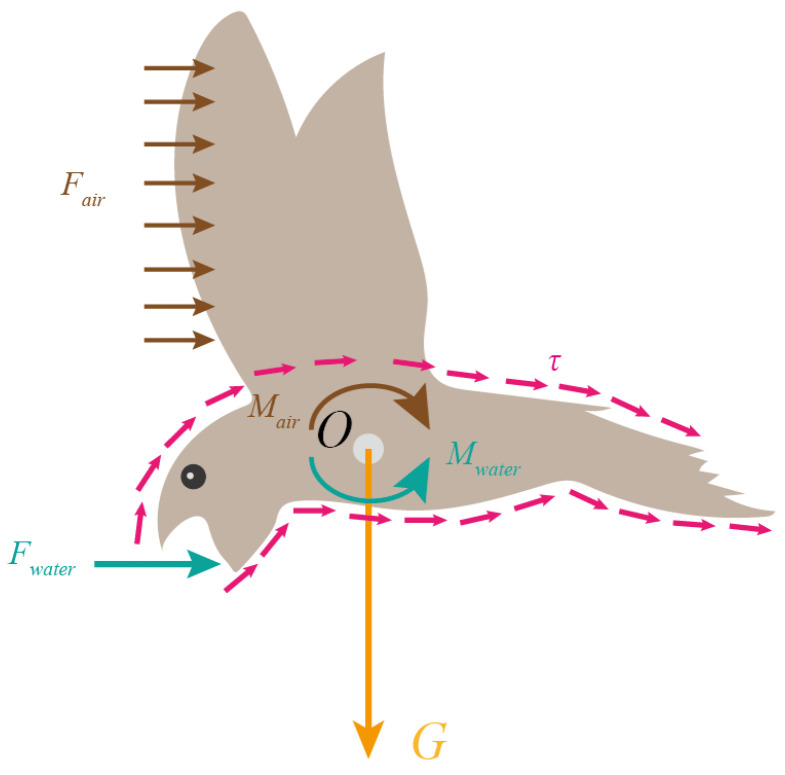
Force analysis of a water-dipping swift.

The drinking behavior of the swift involves intricate force interactions arising from both air and water environments. Illustrated in Figure 6, when the swift dips its beak into water, it experiences three distinct forces: gravity **G**, as well as forces stemming from air and water currents, referred to as $F_{air}$ and $F_{water}$, respectively. Here, $F_{air}$ signifies the aggregate effect of the air’s force upon the swift’s surface, and $F_{water}$ denotes the collective influence of the water’s force on the swift’s surface. Both can be decomposed into normal and parallel components—pressure and friction, respectively—acting on the surface. The pressure and friction attributed to the air are denoted as $F_{d,air}$ and $F_{f,air}$, whereas those originating from the water are expressed as $F_{d,water}$ and $F_{f,water}$. The pressure emerges due to the cumulative effect of the positive pressures *p*, including $p_{air}$ and $p_{water}$, being exerted on the swift’s surface, while the friction emerges from the shear stress τ, comprising $\tau_{air}$ and $\tau_{water}$, acting upon the same surface. This allows $F_{air}$ and $F_{water}$ to be expressed through the following equations:

(1) $$\left\{\begin{array}{c} F_{air}=F_{d,air}+F_{f,air}\\ F_{water}=F_{d,water}+F_{f,water} \end{array}\right.$$

And we arrive at the relationship:

(2) $$F_{air}=\int_{S\_air}\vec{n}\cdot \bar{\tau_{air}^{\prime }}$$

(3) $$F_{water}=\int_{S\_water}\vec{n}\cdot \bar{\tau_{water}^{\prime }}$$

In these equations, the subscripts $s_{air}$ and $s_{water}$ refer to the total aerodynamic and hydrodynamic surface areas of the swift’s body, respectively. Additionally, n represents the unit normal vector of surface elements. $\bar{\tau_{\mathrm{air}}^{\prime }}$ is the viscous tensor of $F_{air}$, and $\bar{\tau_{\mathrm{water}}^{\prime }}$ is the viscous tensor of $F_{water}$. To analyze the moments of forces $F_{air}$ and $F_{water}$ about the center of mass O, we derive expressions for $M_{air}$ and $M_{water}$, respectively, using the following equations:

(4) $$M_{air}=r_{air}\times F_{air}$$

(5) $$M_{water}=r_{water}\times F_{water}$$

Here, $r_{air}$ represents the radial vector corresponding to $F_{air}$, and $r_{water}$ signifies the radial vector pertaining to $F_{water}$. Introducing the concept of residual torque, defined as the disparity between $M_{air}$ and $M_{water}$, is essential at this juncture, it is expressed as $M=M_{water}-M_{air}$.

### 3.3. Water Resistance

**Figure 7:**
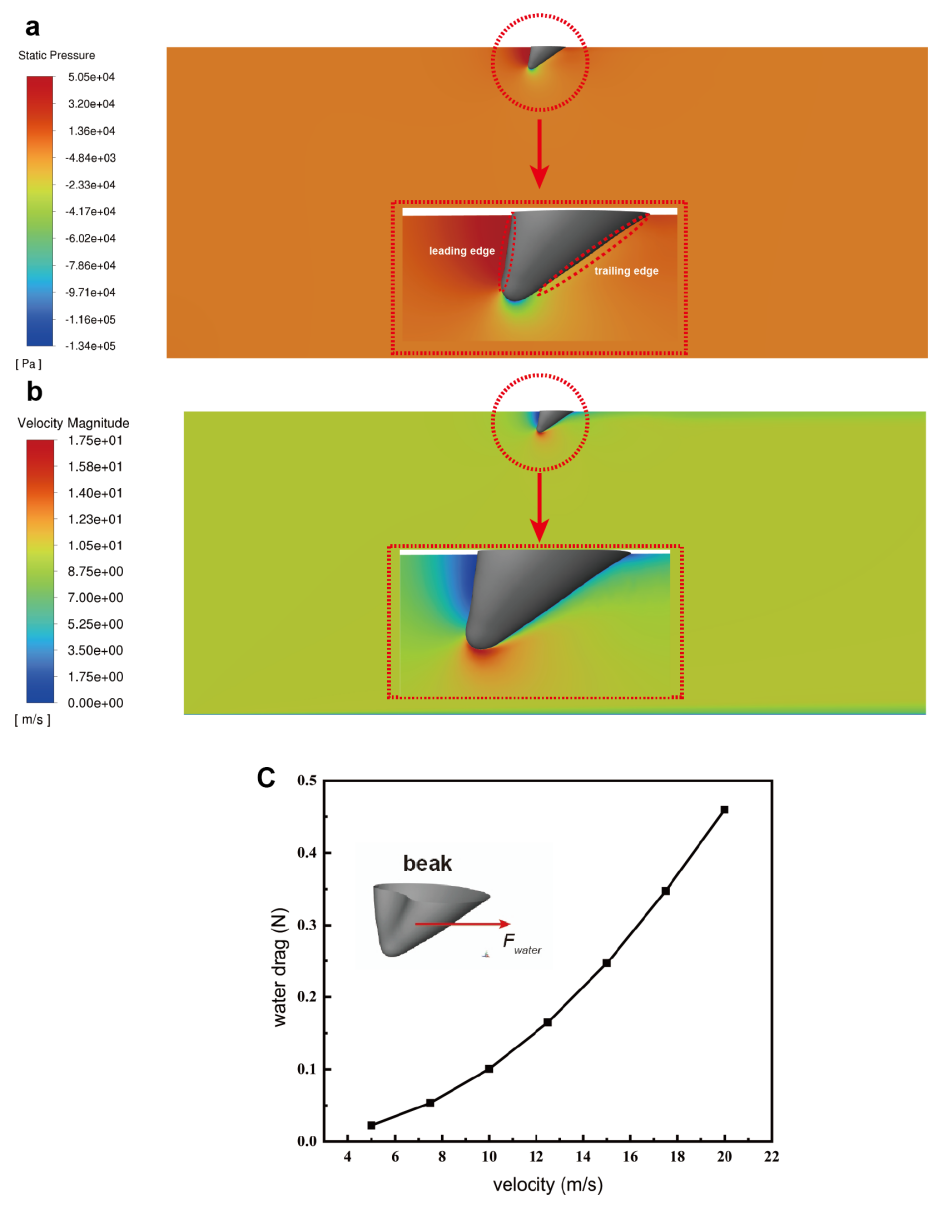
The pressure distribution contour on the beak and the flow field velocity contour while dipping water, along with the water resistance curve experienced by the bird at different velocities. (**a**) Pressure distribution contour on the bird’s beak. (**b**) Velocity distribution contour in the surrounding flow field of the bird’s beak. (**c**) Resistance of water experienced by the bird at different velocities.

During the process of dipping water, the swift’s beak becomes partially submerged, resulting in the emergence of water resistance. In this research, we embarked on simulated calculations aimed at quantifying the water resistance exerted on the swift’s beak while it is immersed in water. Our analysis encompassed the evaluation of forces acting on the beak at seven distinct velocities, namely 5 m/s, 7.5 m/s, 10 m/s, 12.5 m/s, 15 m/s, 17.5 m/s, and 20 m/s. We first considered the case where the velocity is 10 m/s. In this scenario, Figure 7a elucidates the pressure distribution across the swift’s beak, while Figure 7b provides insight into the water flow velocity encompassing the beak. The pressure contour illustrates heightened pressure levels along the leading edge of the beak(Figure 7a), with the trailing surface encountering comparatively lower pressure. A small region of negative pressure is also discernible near the beak’s tip. Shown in Figure 7b, the water flow velocity distribution exhibits the slowest velocity along the beak’s leading surface, while the highest velocity is registered at the tip of the beak. These findings correlate well with the discerned pressure distribution contours. Importantly, pressure and velocity distributions observed at other velocities mirror similar trends to those observed at 10 m/s. Figure 7c portrays the water resistance encountered by the swift across different velocities, a relationship that can be well-fitted by the equation $F_{water}=0.001\;v^{2}$. This also means that within the speed range we considered, the water drag coefficient remains essentially constant. This formulation effectively encapsulates the empirical observations regarding the swift’s water drag at varying speeds.

### 3.4. Air Drag

**Figure 8:**
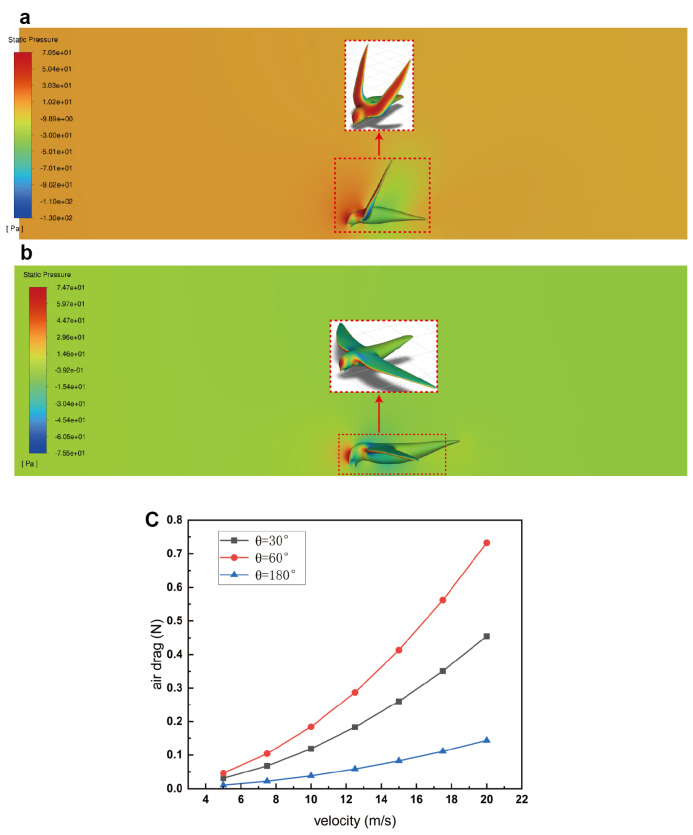
Pressure contour distribution at different wing angles of the swift at a speed of 10 m/s and the variation in air drag at different speeds and wing angles. (**a**) Pressure distribution contour at a velocity of 10 m/s for a wing angle of $60^{\circ }$. (**b**) Pressure distribution contour at a velocity of 10 m/s for a wing angle of $180^{\circ }$. (**c**) The air drag at different velocities and wing angles.

The swift’s interaction with air drag during the process of dipping water can notably impact its overall body stability. Firstly, the water resistance encountered by the beak results in a moment that can destabilize the body, as depicted in Figure 7. However, the concurrent air drag acting upon the wings and body of the swift generates a counteracting moment in the opposite direction. This dynamic force interplay possesses the potential to either mitigate or partially offset the destabilizing effect originating from water resistance. Consequently, a balance in the swift’s body stability is facilitated through this interplay between water resistance and air drag. Secondly, the act of directly immersing the beak into water at elevated flight speeds exposes the swift’s beak to substantial water resistance, which induces a noteworthy destabilizing moment. This mechanical circumstance entails a notable risk for the swift. To mitigate this risk, the swift could potentially adapt by adjusting its wing angles, thereby modulating the air drag and initiating a deceleration process prior to dipping water. In alignment with this hypothesis, Figure 8a,b illustrate the pressure distribution contours surrounding the swift at two distinct wing angles: $60^{\circ }$ and $180^{\circ }$, respectively. Notably, when the wings form an angle of $\theta =180^{\circ }$ (thus adopting a flat-winged configuration), the air drag exerted on the wings is comparatively minimal compared with that experienced at an angle of $\theta =60^{\circ }$. This finding supports the notion that the swift might strategically manipulate its wing angles to regulate air drag and facilitate a controlled deceleration before engaging in water dipping.

Figure 8c elucidates the air drag experienced by the swift across varying velocities and distinct wing angles. Across all velocities considered, a wing angle of $180^{\circ }$ yields the least air drag, whereas a wing angle of $60^{\circ }$ corresponds to the highest drag magnitude. Notably, at low velocities such as the minimum air speed of $v_{air}=5$ m/s, the air drag values for all three wing angles are relatively proximate. Nevertheless, as velocities escalate—e.g., to $v_{air}=20$ m/s—the disparities in air drag become considerably pronounced. Conversely, when analyzing air drag with a consistent wing angle, a consistent trend emerges: air drag escalates in proportion to the velocity. However, the rate of this escalation varies and is contingent on the specific wing angle. Notably, a wing angle of $180^{\circ }$ results in a gradual and moderate rise in drag as velocity increases. Conversely, a wing angle of $60^{\circ }$ exhibits a more substantial increase in air drag in response to augmented velocity. This differential behavior in air drag response underscores the nuanced interplay between wing angles, velocity, and resultant air drag forces. We studied the variation of the air drag coefficient with speed at different angles and concluded that within the speed range we considered, the air drag coefficient slightly decreases with increasing speed. However, this change is very limited. Additionally, the air drag coefficient varies at different angles. The drag coefficient is highest at $\theta =60^{\circ }$, followed by $\theta =30^{\circ }$, and it is lowest at $\theta =180^{\circ }$.

### 3.5. Residual Torque

**Figure 9:**
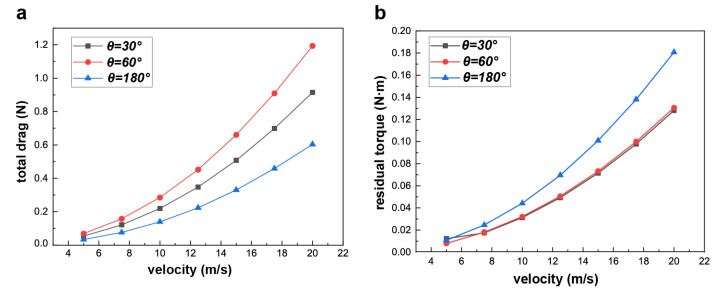
Total drag and residual torque at different velocities and wing angles. (**a**) Total drag at different velocities and wing angles. (**b**) Residual torque at different velocities and wing angles.

As illustrated in Figure 9a, the cumulative drag experienced by the swift exhibits a direct correlation with its flight speed. Consider, for instance, the scenario where the wing angle is $\theta =180^{\circ }$; escalating the flight speed from 5 m/s to 20 m/s translates to an approximate 18-fold surge in the cumulative drag magnitude. It is noteworthy that excessively low flight speeds can lead to stalling during water feeding, posing challenges in maintaining stable aerial motion.

Interestingly, the swift’s water feeding strategy involves flying at a speed of 10 m/s, a tactic that likely serves to avert issues of balance loss. As depicted in Figure 9b, when the swift operates at this velocity, it can strategically diminish its speed by adjusting the wing angle, capitalizing on the augmented flight drag and prepping for water feeding. More specifically, when the swift employs a flat wing configuration ($\theta =180^{\circ }$), the total drag registers at 0.14 N, equivalent to 35% of its body weight. Conversely, by transitioning to a V-shaped wing configuration with $\theta =60^{\circ }$, the total drag encounters an approximate 100% increase, resulting in a noteworthy reduction in the swift’s flight speed. This reduction aligns with the swift’s requirement for a safer and more controlled feeding process.

Subsequently, we proceeded to compute the residual torque that the swift encounters upon making contact with the water surface, as visualized in Figure 9b. Notably, the swift confronts the highest residual torque when its wing angle reaches $180^{\circ }$, a configuration that could elevate the likelihood of the swift falling into the water. In response to this potential risk, the swift possesses the capacity to mitigate its exposure to this destabilizing torque by adjusting its wing angle. For instance, through the strategic reduction of the wing angle from $180^{\circ }$ to $30^{\circ }$, the residual torque experienced by the swift undergoes a substantial decline of 30%. This adaptive modulation of the wing angle effectively functions as an active regulatory mechanism, potentially enhancing the swift’s survival prospects during water feeding scenarios. By minimizing the residual torque through wing angle adjustments, the swift bolsters its stability and increases its chances of maintaining controlled and balanced flight, reducing the risk of inadvertent falls.

## 4. Conclusions

Our study offers insights into the drinking behavior of swifts during flight. We observed that swifts adopt a unique V-shaped wing configuration while feeding on water, which helps maintain stability during the challenging task of water dipping. By autonomously adjusting the angle of the V-shaped wings, swifts fine-tune their body balance, ensuring a successful and well-controlled water feeding process. Through computational fluid dynamics simulations, we analyzed the forces acting on swifts during water dipping. The balance between water resistance and air drag plays a crucial role in their ability to maintain stable flight while drinking. Swifts adjust their flight velocity and wing angles strategically to optimize these forces, enabling them to perform safe and controlled water dips without compromising their stability.

The findings from our research shed light on the flight adaptability of swifts and their exceptional ability to navigate challenging environments. Moreover, the observed wing morphing behavior during water dipping may inspire new designs for next-generation unmanned aerial vehicles with enhanced maneuverability and stability. This study contributes to our understanding of swifts’ aerial feeding strategies and their remarkable adaptations for survival during long-distance flights. It highlights the fascinating interplay between aerodynamics, fluid mechanics, and animal behavior. Our research not only deepens our knowledge of avian flight dynamics but also inspires potential applications in bio-inspired engineering and unmanned aerial vehicle design. Further investigations into the swift flight behavior and their remarkable abilities may lead to innovative advancements in aviation and robotics. The insights gained from studying these agile aerial masters may hold the key to unlocking new frontiers in the field of biomimicry and contribute to the development of more efficient and versatile flight technologies.

## Data Availability

The datasets used or analyzed during the current study are available from the corresponding author upon reasonable request.

## Supplementary Materials

The following supporting information can be downloaded at: https://www.mdpi.com/article/10.3390/biomimetics9080457/s1.
