# Supplementary figures and images for: Swifts Form V-Shaped Wings While Dipping in Water to Fine-Tune Balance
Source: Biomimetics (Basel). 2024 Jul 26;9(8):457. doi: 10.3390/biomimetics9080457 (PMC11351436; doi:10.3390/biomimetics9080457)

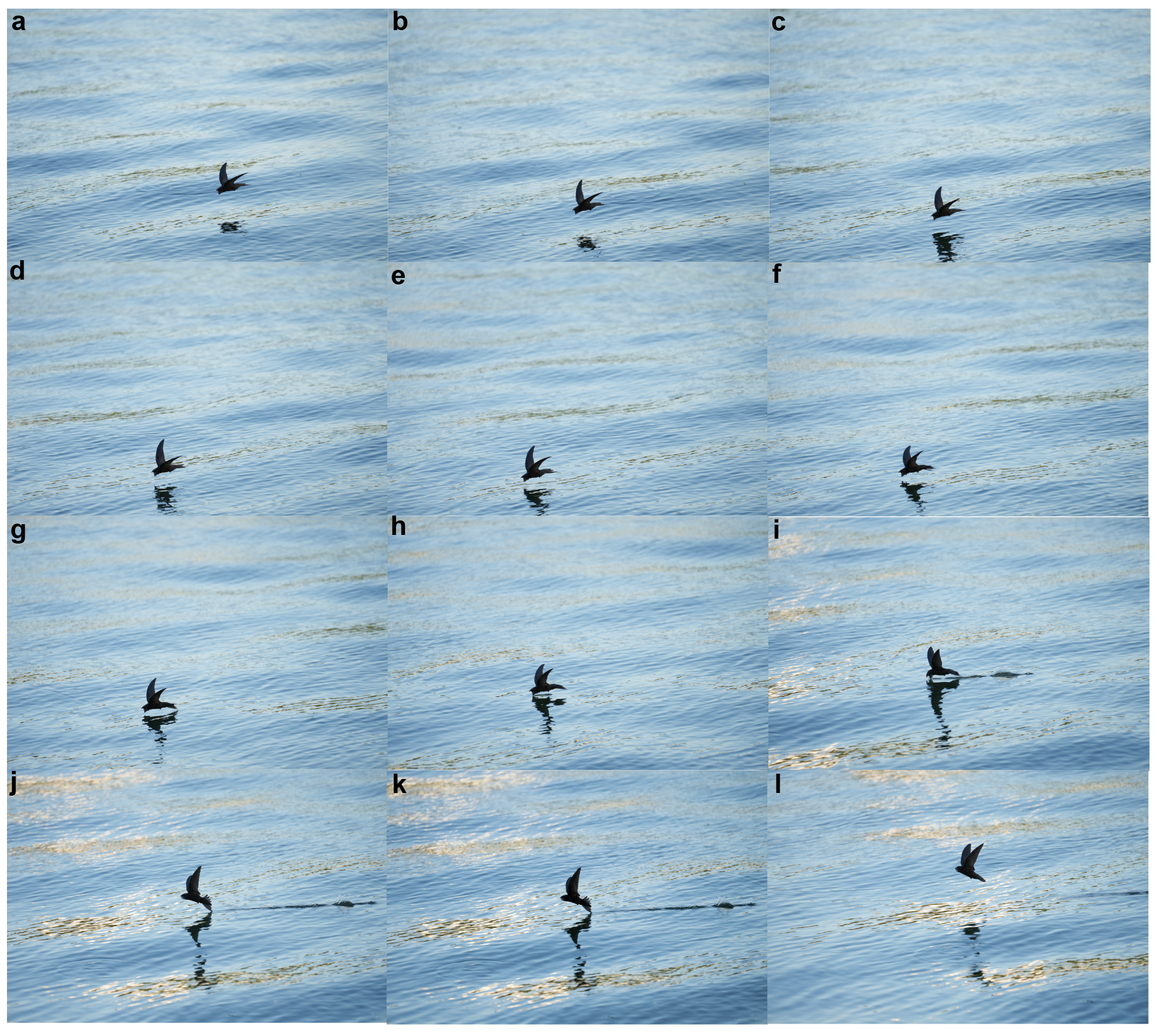

Supplement: Supplementary file 1 [file biomimetics-09-00457-s001.zip › seq1.png]

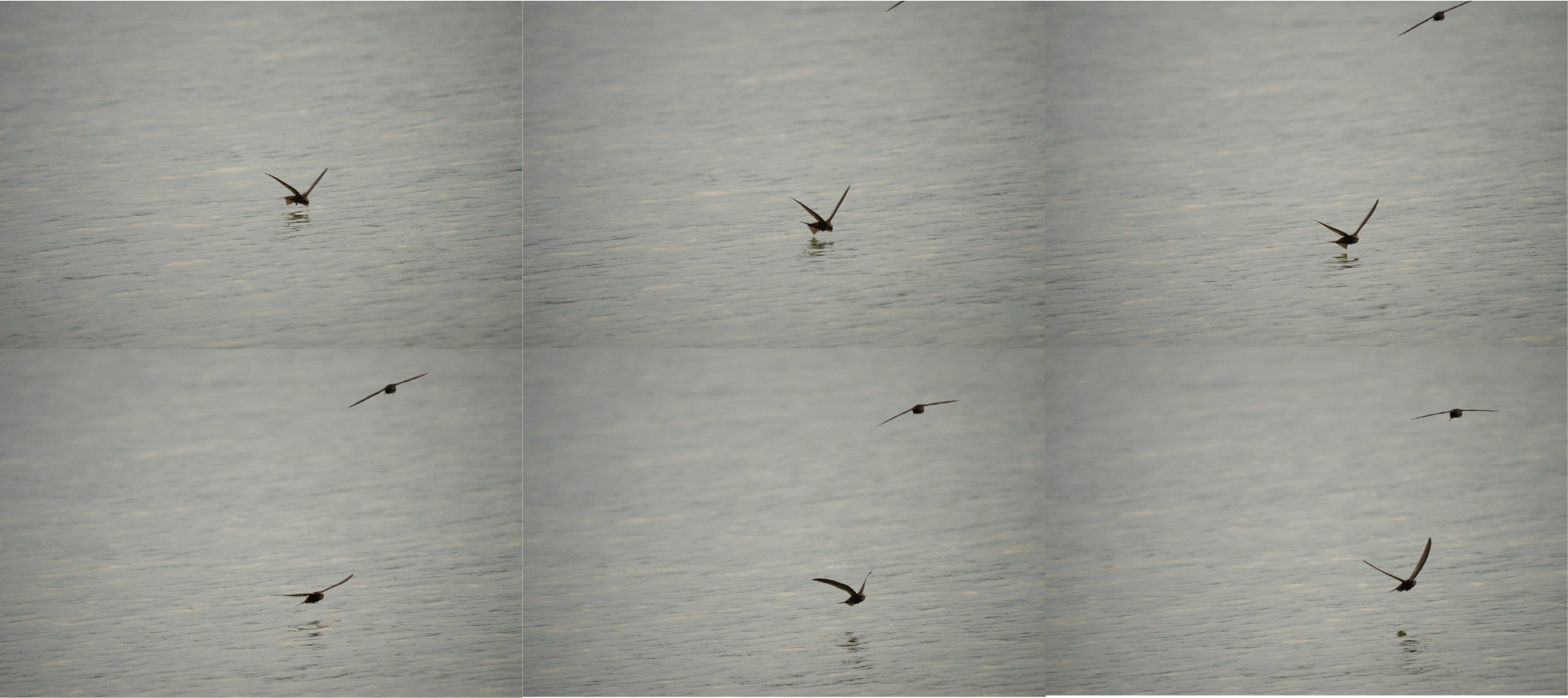

Supplement: Supplementary file 1 [file biomimetics-09-00457-s001.zip › seq10.png]

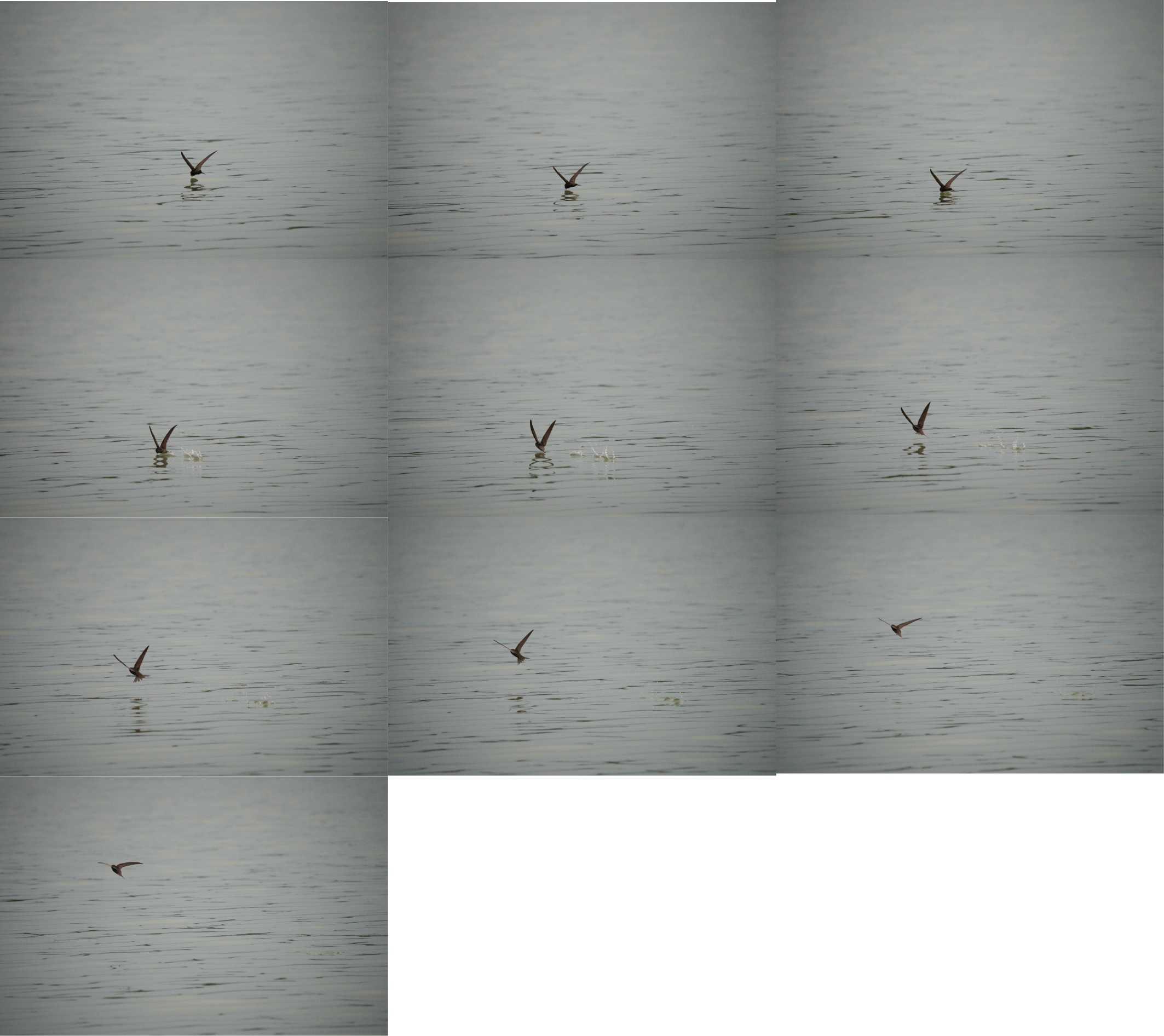

Supplement: Supplementary file 1 [file biomimetics-09-00457-s001.zip › seq11.png]

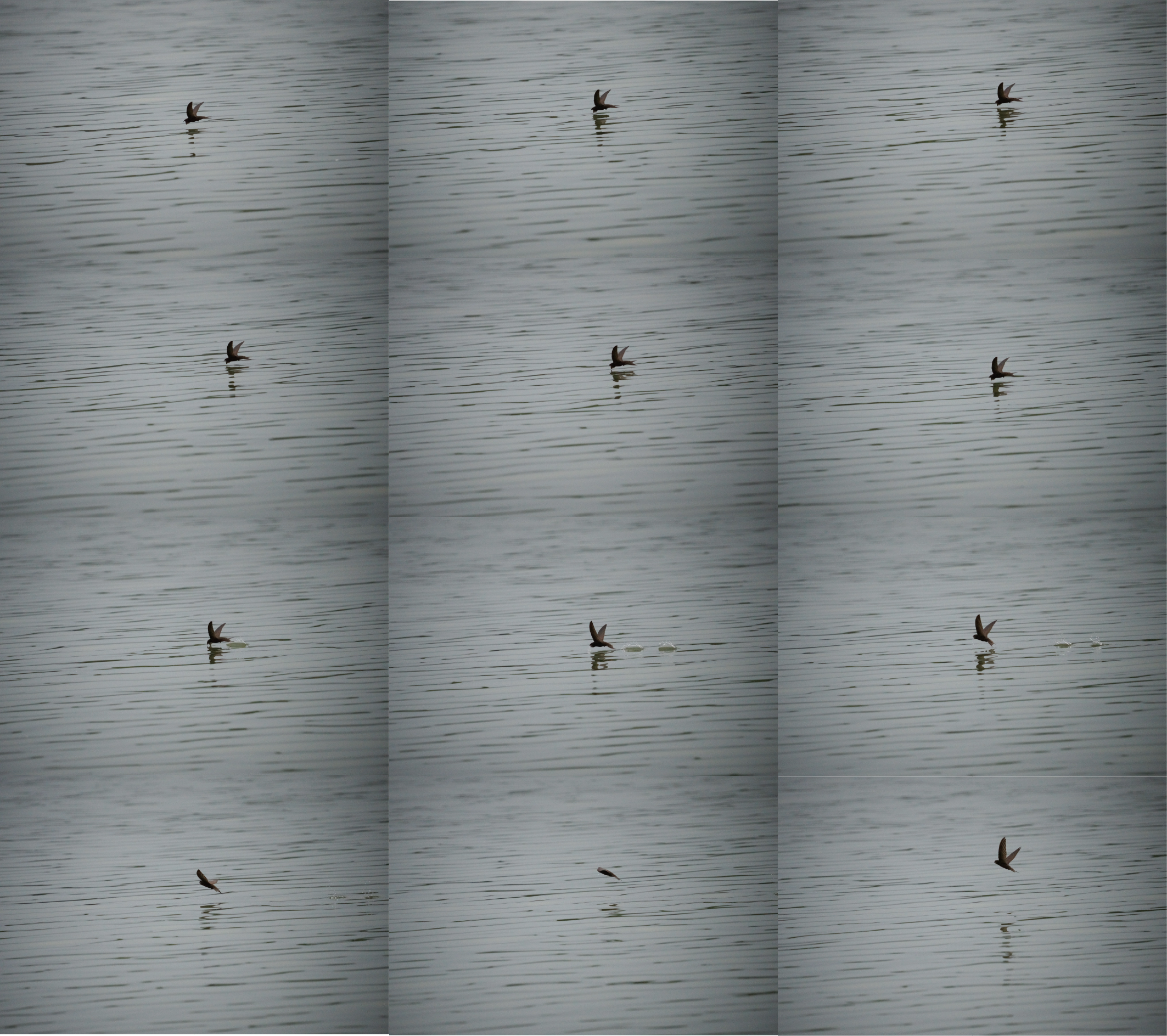

Supplement: Supplementary file 1 [file biomimetics-09-00457-s001.zip › seq12.png]

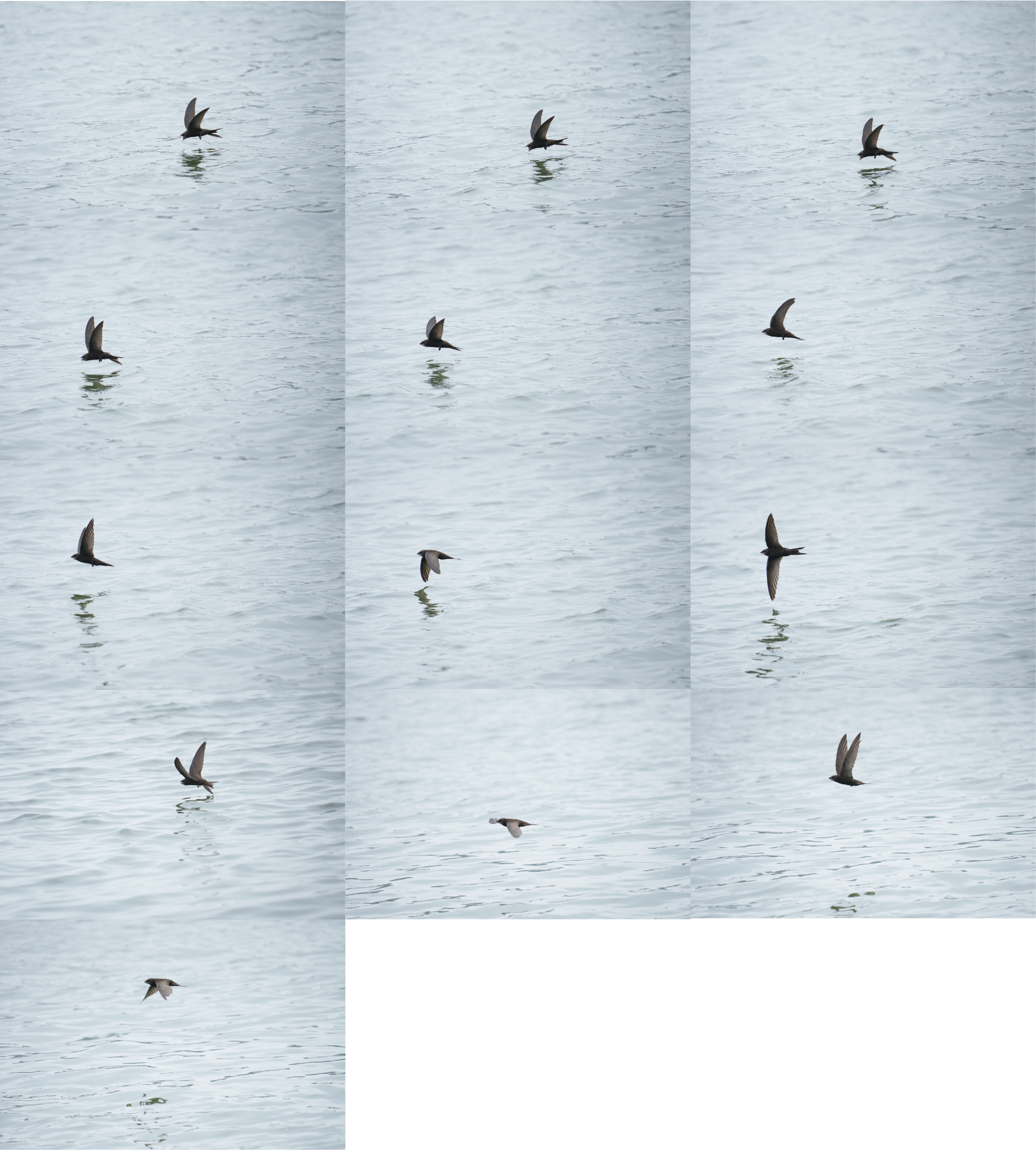

Supplement: Supplementary file 1 [file biomimetics-09-00457-s001.zip › seq13.png]

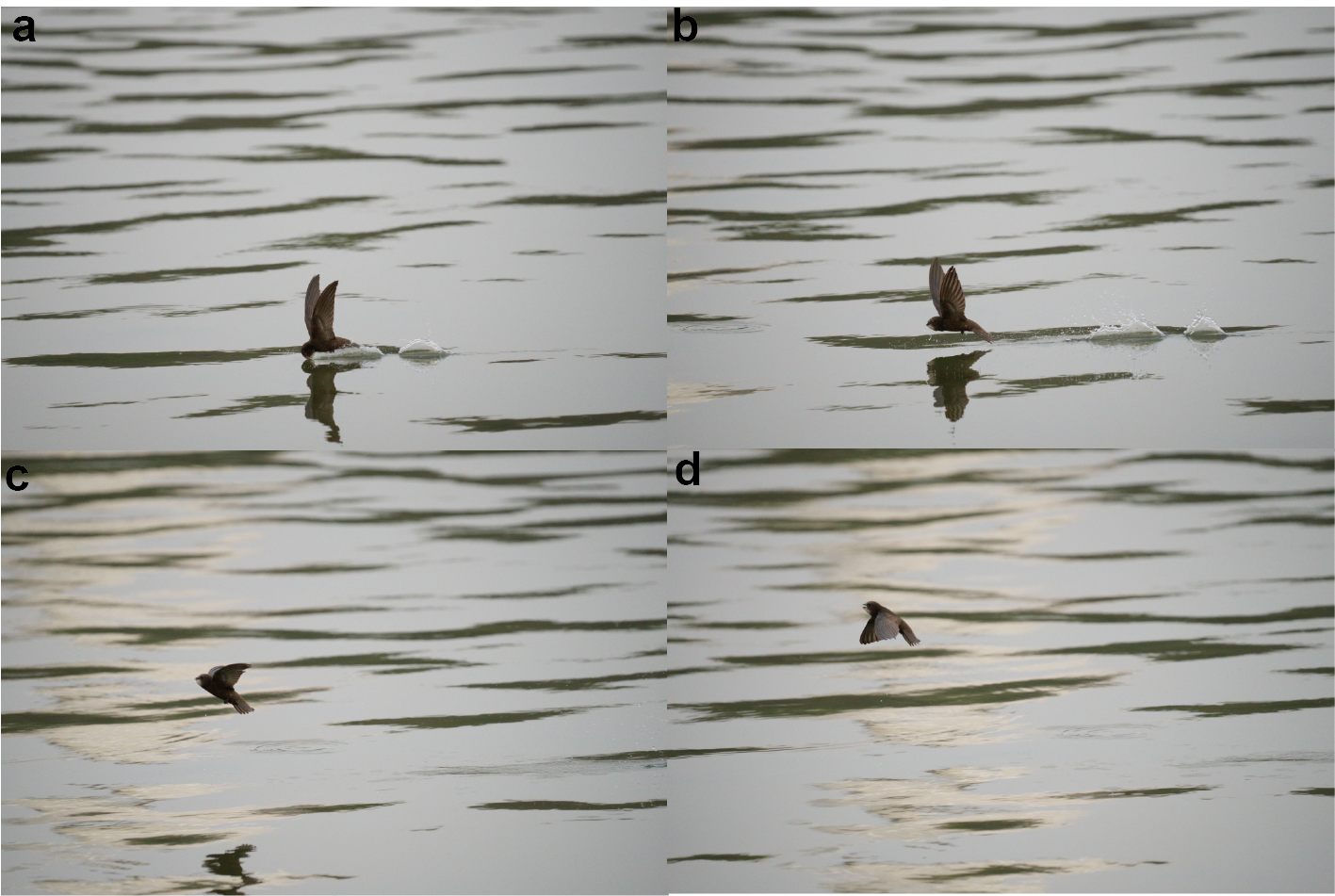

Supplement: Supplementary file 1 [file biomimetics-09-00457-s001.zip › seq2.png]

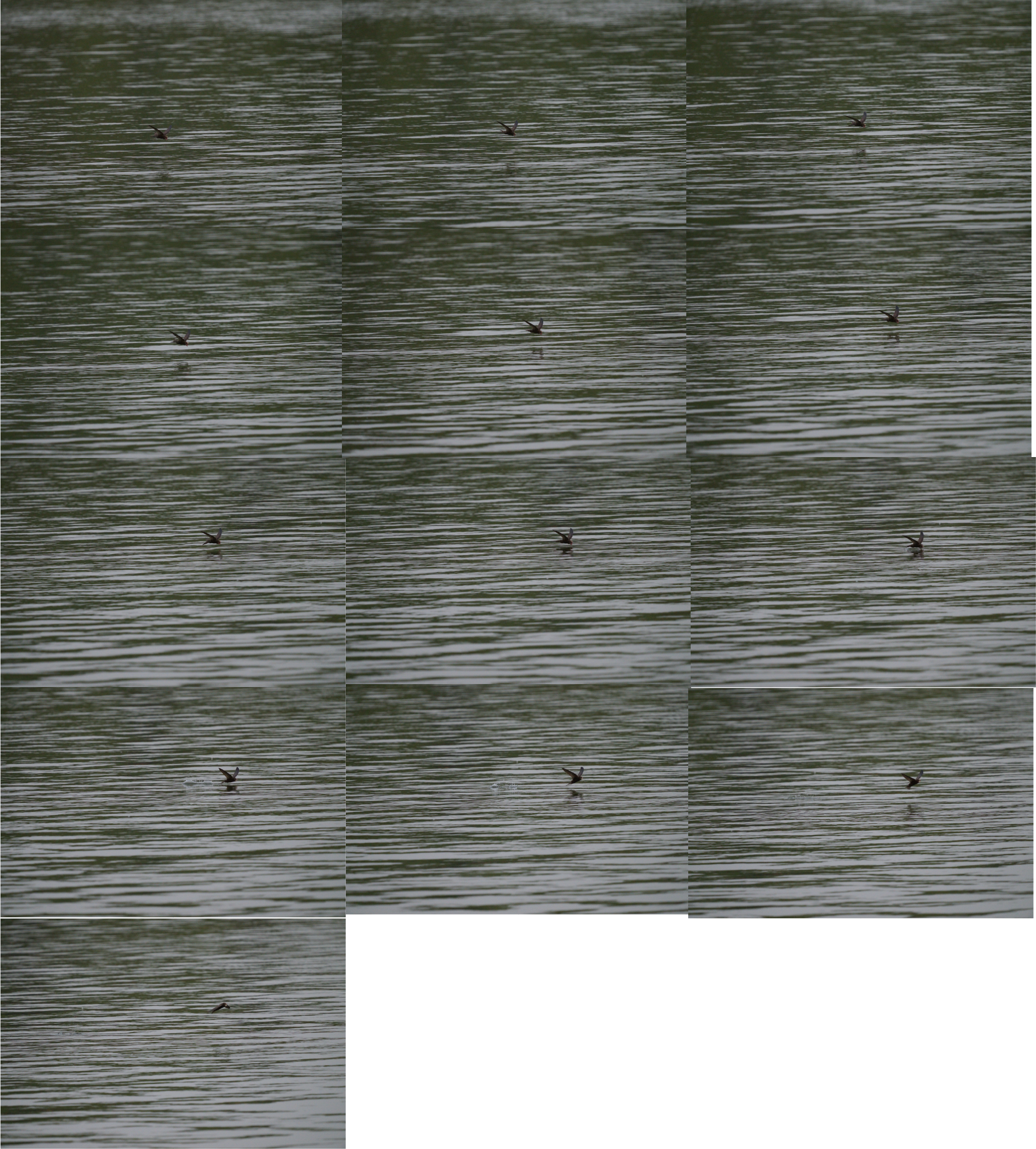

Supplement: Supplementary file 1 [file biomimetics-09-00457-s001.zip › seq3.png]

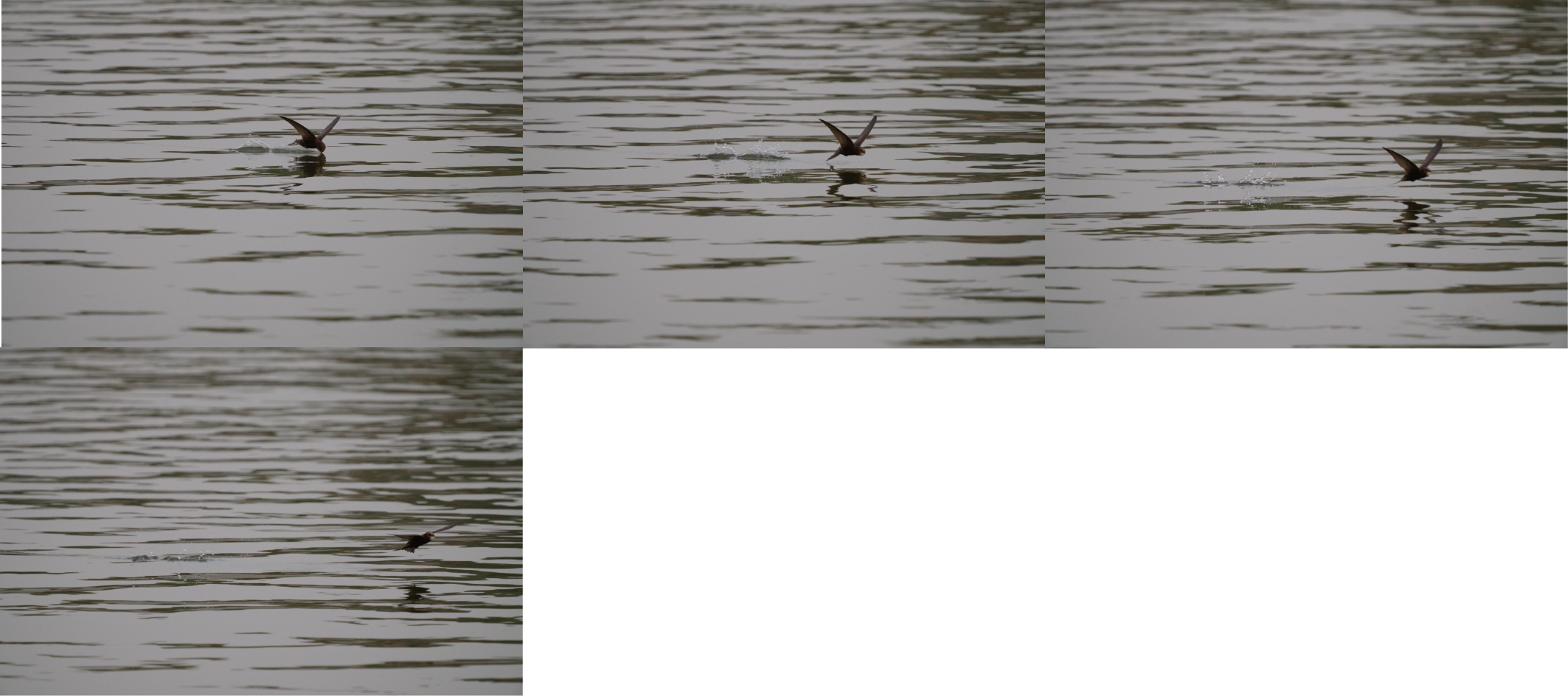

Supplement: Supplementary file 1 [file biomimetics-09-00457-s001.zip › seq4.png]

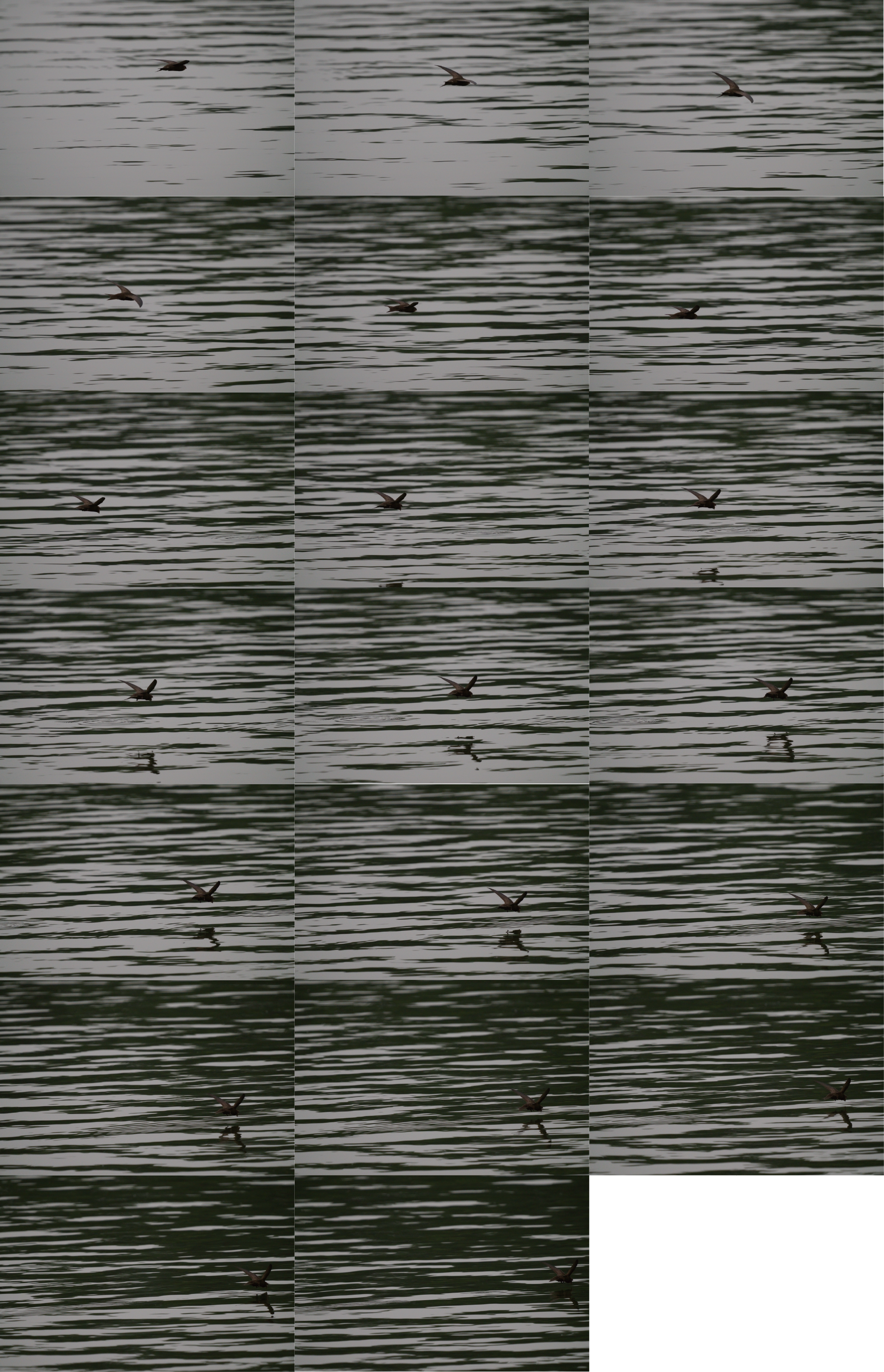

Supplement: Supplementary file 1 [file biomimetics-09-00457-s001.zip › seq5.png]

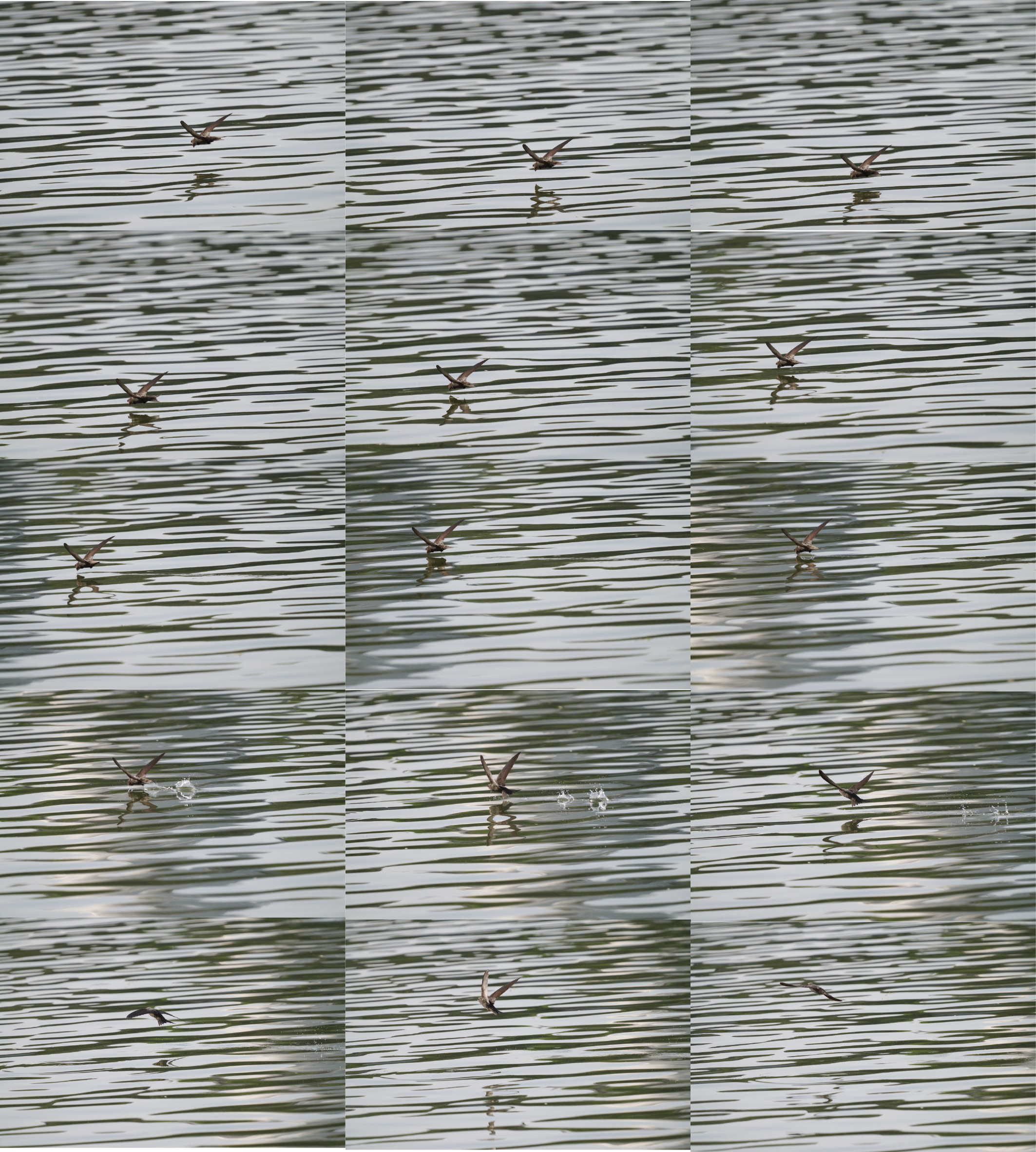

Supplement: Supplementary file 1 [file biomimetics-09-00457-s001.zip › seq6.png]

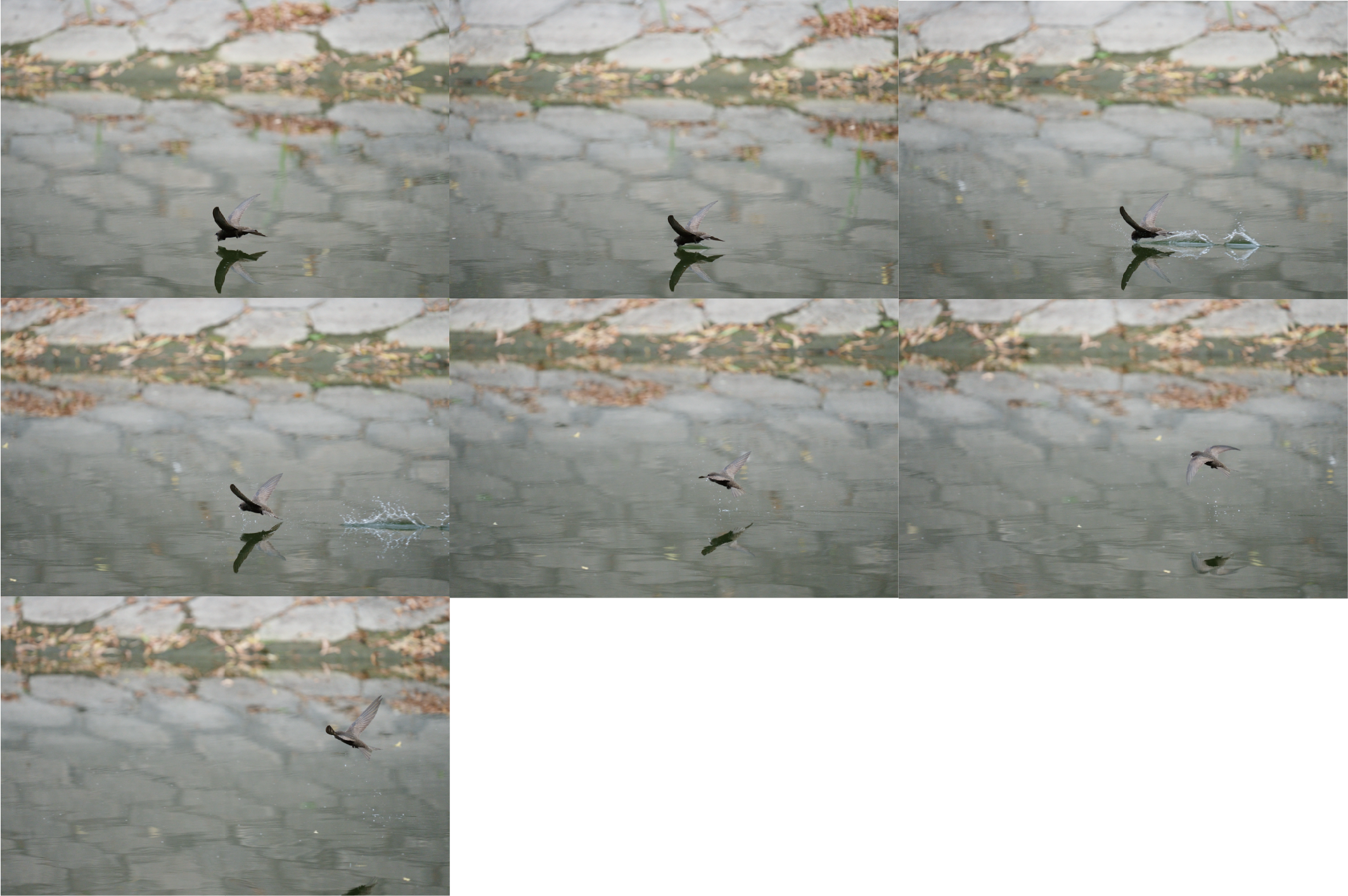

Supplement: Supplementary file 1 [file biomimetics-09-00457-s001.zip › seq7.png]

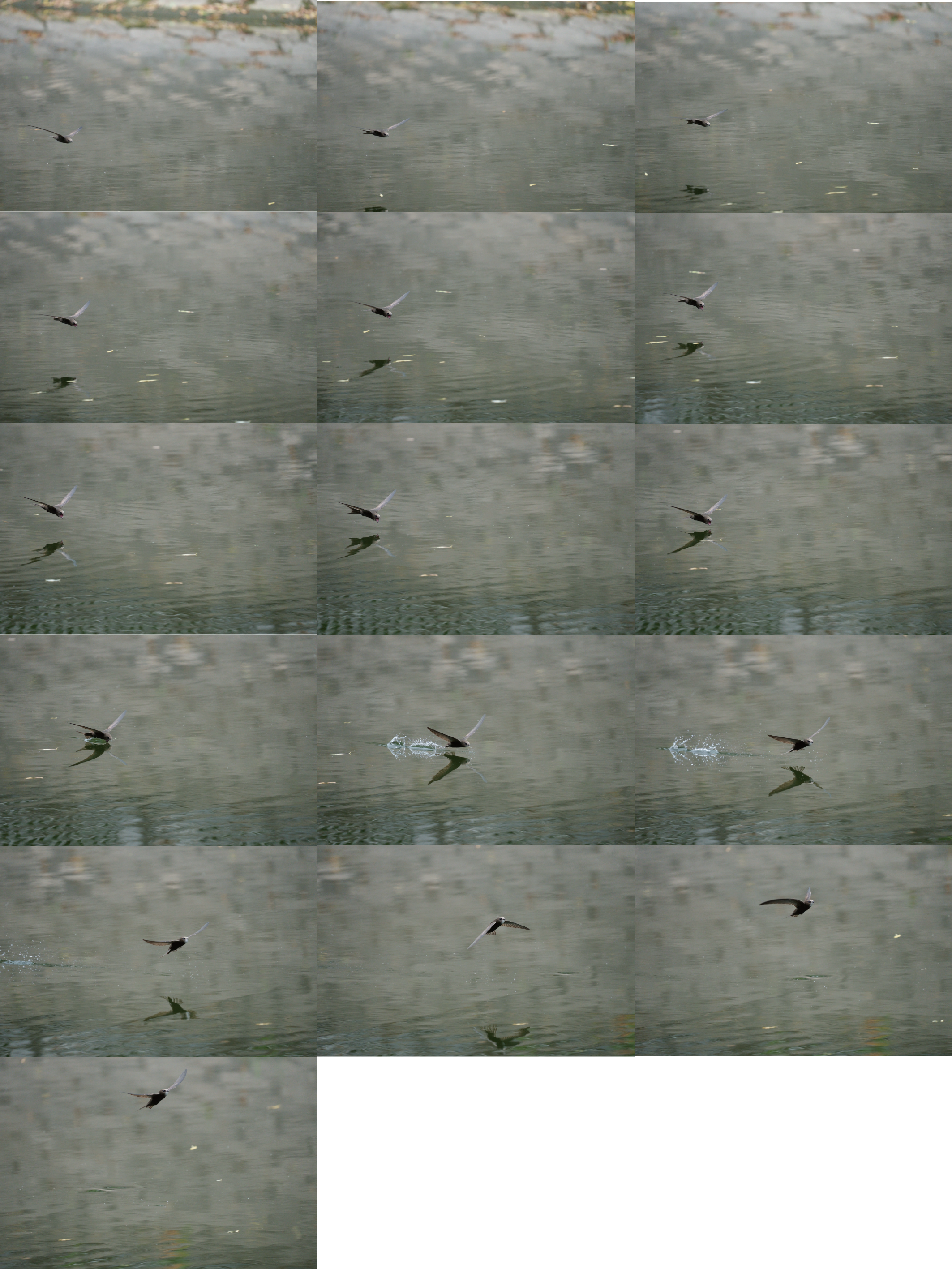

Supplement: Supplementary file 1 [file biomimetics-09-00457-s001.zip › seq8.png]

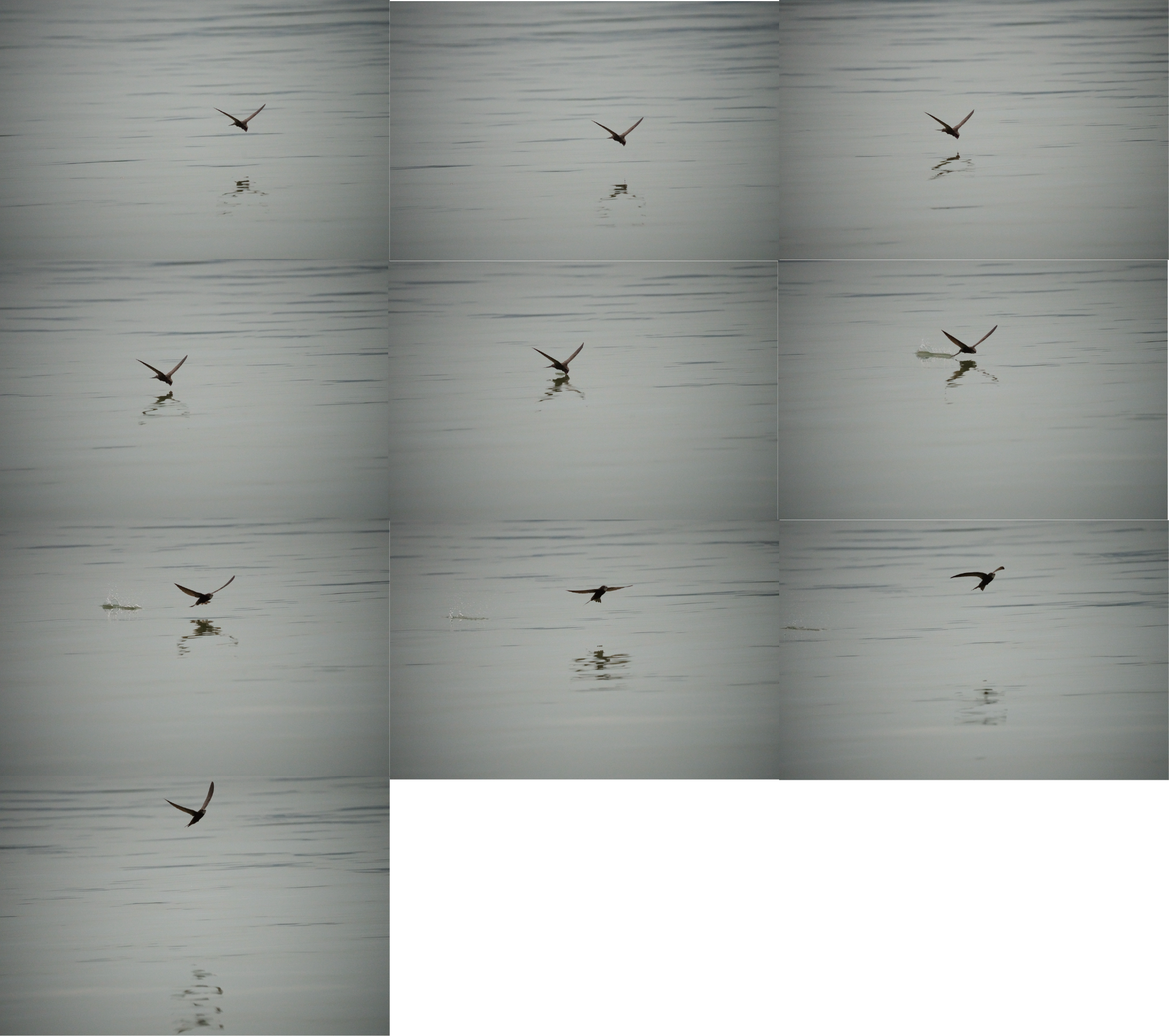

Supplement: Supplementary file 1 [file biomimetics-09-00457-s001.zip › seq9.png]
